# Estimated SARS-CoV-2 infection rate and fatality risk in Gauteng Province, South Africa: a population-based seroepidemiological survey

**DOI:** 10.1093/ije/dyab217

**Published:** 2021-10-30

**Authors:** Portia Chipo Mutevedzi, Mary Kawonga, Gaurav Kwatra, Andrew Moultrie, Vicky Baillie, Nicoletta Mabena, Masego Nicole Mathibe, Martin Mosotho Rafuma, Innocent Maposa, Geoff Abbott, Janie Hugo, Bridget Ikalafeng, Tsholofelo Adelekan, Mkhululi Lukhele, Shabir A Madhi

**Affiliations:** South African Medical Research Council Vaccines and Infectious Diseases Analytics Research Unit, Faculty of Health Sciences, University of the Witwatersrand, Johannesburg, South Africa; Department of Community Health, School of Public Health, Faculty of Health Sciences, University of the Witwatersrand, Johannesburg, South Africa; South African Medical Research Council Vaccines and Infectious Diseases Analytics Research Unit, Faculty of Health Sciences, University of the Witwatersrand, Johannesburg, South Africa; African Leadership in Vaccinology Expertise, Faculty of Health Sciences, University of the Witwatersrand, Johannesburg, South Africa; Department of Clinical Microbiology, Christian Medical College, Vellore, India; South African Medical Research Council Vaccines and Infectious Diseases Analytics Research Unit, Faculty of Health Sciences, University of the Witwatersrand, Johannesburg, South Africa; South African Medical Research Council Vaccines and Infectious Diseases Analytics Research Unit, Faculty of Health Sciences, University of the Witwatersrand, Johannesburg, South Africa; ResearchLinkME, Johannesburg, South Africa; South African Medical Research Council Vaccines and Infectious Diseases Analytics Research Unit, Faculty of Health Sciences, University of the Witwatersrand, Johannesburg, South Africa; South African Medical Research Council Vaccines and Infectious Diseases Analytics Research Unit, Faculty of Health Sciences, University of the Witwatersrand, Johannesburg, South Africa; Division of Epidemiology and Biostatistics, School of Public Health, Faculty of Health Sciences, University of the Witwatersrand, Johannesburg, South Africa; Community Oriented Primary Care Research Unit, Department of Family Medicine, University of Pretoria, Pretoria, South Africa; Community Oriented Primary Care Research Unit, Department of Family Medicine, University of Pretoria, Pretoria, South Africa; Gauteng Department of Health, Johannesburg, South Africa; Gauteng Department of Health, Johannesburg, South Africa; The Division of Orthopedics, University of Witwatersrand, Johannesburg, South Africa; South African Medical Research Council Vaccines and Infectious Diseases Analytics Research Unit, Faculty of Health Sciences, University of the Witwatersrand, Johannesburg, South Africa; African Leadership in Vaccinology Expertise, Faculty of Health Sciences, University of the Witwatersrand, Johannesburg, South Africa

**Keywords:** COVID-19, SARS-CoV-2, coronavirus, seroprevalence, serosurvey, infection-mortality risk

## Abstract

**Background:**

Limitations in laboratory testing capacity undermine the ability to quantify the overall burden of severe acute respiratory syndrome coronavirus-2 (SARS-CoV-2) infection.

**Methods:**

We undertook a population-based serosurvey for SARS-CoV-2 infection in 26 subdistricts, Gauteng Province (population 15.9 million), South Africa, to estimate SARS-CoV-2 infection, infection fatality rate (IFR) triangulating seroprevalence, recorded COVID-19 deaths and excess-mortality data. We employed three-stage random household sampling with a selection probability proportional to the subdistrict size, stratifying the subdistrict census-sampling frame by housing type and then selecting households from selected clusters. The survey started on 4 November 2020, 8 weeks after the end of the first wave (SARS-CoV-2 nucleic acid amplification test positivity had declined to <10% for the first wave) and coincided with the peak of the second wave. The last sampling was performed on 22 January 2021, which was 9 weeks after the SARS-CoV-2 resurgence. Serum SARS-CoV-2 receptor-binding domain (RBD) immunoglobulin-G (IgG) was measured using a quantitative assay on the Luminex platform.

**Results:**

From 6332 individuals in 3453 households, the overall RBD IgG seroprevalence was 19.1% [95% confidence interval (CI): 18.1–20.1%] and similar in children and adults. The seroprevalence varied from 5.5% to 43.2% across subdistricts. Conservatively, there were 2 897 120 (95% CI: 2 743 907–3 056 866) SARS-CoV-2 infections, yielding an infection rate of 19 090 per 100 000 until 9 January 2021, when 330 336 COVID-19 cases were recorded. The estimated IFR using recorded COVID-19 deaths (*n* = 8198) was 0.28% (95% CI: 0.27–0.30) and 0.67% (95% CI: 0.64–0.71) assuming 90% of modelled natural excess deaths were due to COVID-19 (*n* = 21 582). Notably, 53.8% (65/122) of individuals with previous self-reported confirmed SARS-CoV-2 infection were RBD IgG seronegative.

**Conclusions:**

The calculated number of SARS-CoV-2 infections was 7.8-fold greater than the recorded COVID-19 cases. The calculated SARS-CoV-2 IFR varied 2.39-fold when calculated using reported COVID-19 deaths (0.28%) compared with excess-mortality-derived COVID-19-attributable deaths (0.67%). Waning RBD IgG may have inadvertently underestimated the number of SARS-CoV-2 infections and conversely overestimated the mortality risk. Epidemic preparedness and response planning for future COVID-19 waves will need to consider the true magnitude of infections, paying close attention to excess-mortality trends rather than absolute reported COVID-19 deaths.

Key Messages

- There was marked heterogeneity in seroprevalence across subdistricts ranging from 5.5% to 43%; the overall seroprevalence was 19.1% and did not differ by stratified age groups.
- Overall, we estimate that there were 2.89 million SARS-CoV-2 infections compared with 332 000 reported COVID-19 cases that had been documented in Gauteng at the end of the serosurvey, resulting in a 2- to >20-fold difference between the calculated SARS-CoV-2 infections and the recorded COVID-19 cases across the subdistricts.
- The calculated mortality risk was 0.28% using recorded Covid-19 deaths and 0.68% assuming that 90% of excess mortality due to non-accidental deaths were due to COVID-19, both of which is the lower range reported in a recent meta-analysis that mainly included data from high-income countries. Nevertheless, our calculated mortality rates of 1197–1379 (using excess-mortality-derived COVID-attributable deaths) per million of the population are among the highest reported globally.
- Notably, we observed that 53% of individuals with past self-reported polymerase-chain-reaction-confirmed COVID-19 were seronegative, possibly due to the waning of the IgG antibody that might have occurred following mild COVID-19. Implications of this include that we inadvertently underestimate past SARS-CoV-2 infections and possibly overestimate mortality risk by a factor of 2.

## Introduction

Africa’s population of 1.3 billion constitute 18% of the global population (*n* = 7.8 billion).^1^ Nevertheless, only 2.1% (3 176 707 of 151 803 822) and 2.6% (82 870 of 3 186 538) of documented coronavirus disease (COVID-19) cases and deaths, respectively, as of 30 April 2021 have been recorded in Africa.^2^ Furthermore, South Africa, which constitutes 4.5% (*n* = 59.6 million) of Africa’s population, accounts for 50% (1 582 842/3 176 707) and 66% (5406/82 870) of Africa’s officially documented COVID-19 cases and deaths, respectively. Constraints in accessing healthcare, including limited laboratory quality and infrastructure to test for severe acute respiratory syndrome coronavirus-2 (SARS-CoV-2) infection, likely led to underestimates of the burden of SARS-CoV-2 in Africa.^3^ South Africa has the highest cumulative testing rate (total number of tests per 1000 people) for SARS-CoV-2 infections (179 per 1000 population as of 29 April 2021) in Africa, although it lags behind the testing rates in high-income countries such as the USA (1249) and the UK (2232),^4^ indicating more than one test per individual.

Understanding the force of SARS-CoV-2 infection and the burden of COVID-19 at a country level is essential to inform future planning and management of the ongoing COVID-19 pandemic, including the deployment of COVID-19 vaccines. Population-based seroepidemiological surveys of past SARS-CoV-2 infection could assist in delineating the number of past infections and the infection-mortality risk, and determining the ongoing susceptibility of communities to COVID-19.^5–7^ Recent systematic reviews on SARS-CoV-2 serosurveys have demonstrated a paucity of well-conducted studies, particularly from low- and middle-income settings, and variation in the population groups sampled.^8–10^ Most of these surveys were based on convenience samples such as blood donors and healthcare workers or focused on high-risk areas and thus were not representative of the general population. Chen *et al.* reported that only 2.0% (8/404) of the serosurveys undertaken until 22 December 2020 were in African settings, of which two sampled the general population in Niger State (Nigeria)^11^ and Addis Ababa (Ethiopia)^12^ in which the seroprevalence was 25.4% and 8.8%, respectively. A more recent population-based serosurvey in Zambia reported a seroprevalence of 10.6% (range: 6.0–14.4%) through to 27 July 2020.^13^ Another from Juba, South Sudan, reported a seroprevalence of 38.5% (range: 30.1–60.6%) through to September 2020.^14^ The details of the 4 community-based and 18 non-population-based serosurveys in Africa are summarized in Supplementary Table S1 (available as Supplementary data at *IJE* online).

The primary objective of this survey was to determine the seroprevalence of the SARS-CoV-2 receptor-binding domain (RBD) immunoglobulin-G (IgG) in each of 26 subdistricts, nested in 5 districts, in Gauteng Province (population 15.9 million), South Africa. Secondary objectives included triangulating the calculated number of SARS-CoV-2 infections based on the serosurvey with officially recorded COVID-19 deaths^15^ and excess mortality from natural causes (obtained from the National Population Register)^16^ to estimate the number of SARS-CoV-2 infections and mortality risks. We analysed the characteristics associated with seropositivity.

## Methods

### Study setting

Gauteng, the ‘economic hub’ and one of nine South African provinces, is demarcated into five health districts constituting 26 subdistricts. Gauteng constitutes 1.5% of South Africa’s landmass of 18 178 square kilometres (km^2^), but 26% (15.9/59.6 million) of its population. The overall population density (people per square kilometre) in Gauteng is 737, ranging from <10 to 63 211 by municipality. The City of Johannesburg district (population density 3400 people/km^2^) ranks among the top 10 most densely populated cities globally.^17^ The epidemiology of COVID-19 in Gauteng and government responses are detailed in Supplementary Text 1.1 (available as Supplementary data at *IJE* online).

The serosurvey in Gauteng was initiated on 4 November 2020, approximately 8 weeks after the SARS-CoV-2 nucleic acid amplification test (NAAT) positivity for suspected SARS-CoV-2 infection had declined to <10% after the peak of the first COVID-19 wave. A subsequent NAAT test positivity of >10% was used as a proxy for defining the onset of the resurgence in Gauteng. The last sampling was on 22 January 2021, 9 weeks after the onset of the SARS-CoV-2 resurgence in Gauteng.

### Sample size and sampling

The sample-size calculation was based on the Africa Centres for Disease Control and Prevention Generic protocol for a population-based, age- and gender-stratified serosurvey study for SARS-CoV-2^18^ and on the World Health Organization’s population-based age-stratified seroepidemiological investigation protocol for COVID-19 virus infection.^5^ Assuming a seroprevalence of 10%, a response rate of 0.75, intra-cluster correlation (ICC)of 0.33, with a precision of 0.1, α of 0.05 and design effect of 3.31, the resultant required overall survey sample size was 6025 (61–1948 by subdistrict) individuals. The design effect of 3.31 was based on the observation that the seroprevalence rates vary by geographic area within the same region.^18^ A conservative ICC of 0.33 was used considering the clustering nature of respiratory diseases.

Three-stage sampling was employed. First, the GeoTeraImage (GTI)-2019 data set (https://geoterraimage.com/), which comprises >16 000 ‘small areas’ used for demarcating census areas, was stratified by housing type. Systematic random cluster selection without replacement and probability proportional to the estimated size was then used to select clusters. Finally, a random sample of nine households was selected from each cluster. The number of clusters, hence the households selected per subdistrict, was proportional to the subdistrict population size. The average household size was assumed to be four individuals based on census data with a target of nine households per cluster. All individuals residing in sampled households, irrespective of age, were eligible. Demographic and epidemiologic data were collected using an electronic questionnaire.

### Specimen collection and laboratory methods

A dried blood spot (DBS) was obtained by finger prick using a single-use lancet needle for pricking of the finger, with 3–5 DBS collected on PerkinElmer 226, PerkinElmer Health Sciences, Inc. filter cards for each individual. The RBD IgG was measured by quantitative assay on the Luminex platform. The assay reference serum was calibrated against research reagent NIBSC 20/130 distributed by the National Institute for Standards and Biological Control (NIBSC) (NIBSC, Potters Bar, UK, https://www.nibsc.org/).

The DBS were dried for 3 hours at room temperature, then packed into plastic pouches with silica-gel sachets and stored at –20ºC until analysis. For analysis, a 6-mm hole punch was cut from the filter card and added to 600 μl of assay buffer, assuming 6 μl of serum in each 6-mm blood spot and the final 1/100 dilution was achieved. The spot was kept in a shaker at 2–8ºC overnight for elution and the following day centrifuged at 2000 *g* for 10 minutes before analysis. Significant correlation between the paired serum and the DBS was observed with an *r* value of 0.935 for the RBD IgG. The expression plasmid encoding for SARS-CoV-2 RBD was obtained from the Florian Krammer, Mount Sinai, USA, and expressed as described.^19^ Quantitative RBD IgG antibody concentrations were measured using a bead-based assay on the singleplex Luminex platform. RBD protein was coupled to the magnetic microsphere beads (Bio-Rad, USA) using a two-step carbodiimide reaction. An in-house reference serum was developed by pooling convalescent serum from adults with SARS-CoV-2 infection confirmed by NAAT. This interim reference serum was calibrated against research reagent NIBSC 20/130 distributed by the National Institute for Standards and Biological Control (NIBSC) (NIBSC, Potters Bar, UK, https://www.nibsc.org/). The binding antibody unit (BAU) values assigned to the in-house reference serum for RBD IgG was 1242 BAU/mL. Serum samples collected prior to 2020 (*n* = 31) were used for the analysis of assay specificity; 26 BAU/mL was selected as the threshold indicative of seropositivity for SARS-CoV-2 RBD IgG, based on the highest value of RBD IgG in samples from the pre-COVID-19 era. The sensitivity of the assay in detecting past or current SARS-CoV-2 infection was assessed using serum samples obtained from 15 randomly selected asymptomatic, mild or moderate COVID-19 cases confirmed by SARS-CoV-2 NAAT testing, who had serial sampling undertaken prior to and after symptom onset. The sensitivity of the RBD IgG assay was 75% for samples taken 7–14 days and 100% for samples taken ≥14 days after testing positive for SARS-CoV-2 on NAAT. Bead fluorescence was read using the Bio-Plex 200 instrument (Bio-Rad) using Bio-Plex manager5.0.

### Statistical analyses

SARS-CoV-2 seroprevalence was calculated as the percentage of the sampled population that had SARS-CoV-2 antibodies. By applying the seroprevalence to the 2020 mid-year population estimates^17^ at subdistrict, district and provincial levels, we estimated the number of SARS-CoV-2 infections at the population level from the start of the pandemic to the time of serosurvey. National COVID-19 surveillance is done through the National Institute of Communicable Diseases;^15^ we compared reported COVID-19 numbers with seroprevalence to estimate the difference between reported COVID-19 cases and actual SARS-CoV-2 infections.

The case-fatality ratio (CFR) was computed as the percentage of reported COVID-19 cases resulting in death. A crude COVID-19-mortality rate per million population was initially computed using the number of reported COVID-19 deaths. To account for the under-reporting of COVID-19 deaths, we adjusted the mortality rate per million population using excess mortality due to natural causes since the onset of the pandemic by assuming that 90% of the excess mortality was due to COVID-19. The infection fatality rate (IFR) was calculated as the percentage of reported COVID-19 deaths from the calculated SARS-CoV-2 infections. Further, we calculated an adjusted IFR assuming that 90% of the excess natural mortality was due to COVID-19. The excess-mortality data were obtained from the South Africa Medical Research Council modelling of the number of excess deaths from natural causes above the upper prediction level based on historical data.^16^ There was strong concordance in the trajectory of documented COVID-19 deaths and excess mortality in South Africa, including in Gauteng.^16^ We used COVID-19-case data through to 9 January 2021 rather than the end of the sampling period (22 January 2021) due to the possible lag of 7–14 days in IgG responses following primary SARS-CoV-2 infection.^19–21^ As of 9 January 2021 in Gauteng, there were 21 582 excess natural deaths in people >1 year of age compared with 6142 recorded COVID-19 deaths.^22^

Stata (version 16.1) was used for survey data analyses with sampling weights calculated based on the sampling frame. The data set was set as the survey data, using the survey set command in Stata, prior to analysis. Descriptive statistics inclusive of frequencies, percentages, means and medians were computed and compared using Pearsons χ^2^ for categorical variables, and *t*-tests and the median test for continuous variables. Factors associated with seropositivity were determined in an adjusted logistic-regression model, accounting for clustering at the household level. All factors that were significant at *p* < 0.15 from univariate analysis were included in the adjusted model.

## Results

### Survey-population description

**Figure 1:**
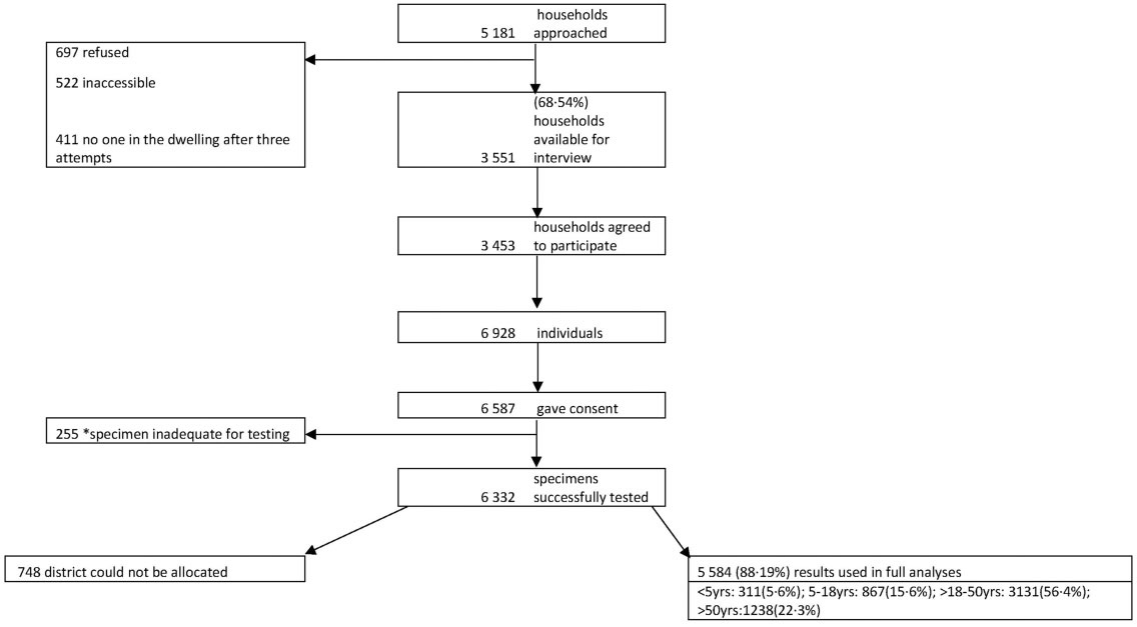
Flow of households and participants included in the seroprevalence survey. We illustrate the flow of participants included in survey analyses from approaching the households to negotiate participation through to specimen collection and processing. Absolute numbers are presented. The final analysis included 5584 individuals in 26 subdistricts. *Inadequate specimen refers to dried blood spots with insufficient filter-paper saturation and hence low specimen yield for serology testing.

Overall, 5181 households were visited. Ninety-seven percent (*n* = 3453) of 3551 accessible households participated in the survey. The median household occupancy was 2 [interquartile range (IQR): 2–4]. Overall, there were 6928 individuals in the participating households, 6587 (95.1%) who consented to provide a DBS (Figure 1). The median age of participants was 34 years (IQR: 21–48 years), including 311 (5.6%) <5 and 647 (11.7%) >60 years of age. Seventeen per cent (906/5232) of the sampled individuals resided in informal dwellings. Among adults (>18 years old; *n* = 4406), 57% (*n* = 2511) were unemployed, 5.2% (*n* = 227) were students and 3.6% (*n* = 160) were healthcare workers (Table 1). Self-reported underlying illnesses in individuals >18 years of age included current or past tuberculosis (27.3%), diabetes (26.5%), hypertension (22.5%) and HIV (16.5%). Also, 864 (21.1%) and 219 (5.4%) of adults had one or at least two underlying chronic medical illnesses (Table 1).

**Table 1:** Demographic characteristics and association with SARS-CoV-2 seropositivity

	*N*	Percentage	Seroprevalence	Unadjusted odds ratio (95% CI)	Adjusted odds ratio (95% CI)
Gender					
Male	2244	40.2	16.9% (15.4–18.5)	Ref.	Ref.
Female	3331	59.7	20.6% (19.3–22.0)	1.28 (1.11–1.47)	1.24 (1.06–1.45)
Age (years) (median; IQR)	34	21–48			
Age categories (years)					
<5	311	5.6	18.0% (14.1–22.7)	0.93 (0.68–1.26)	0.99 (0.71–1.39)
5–18	867	15.6	17.2% (14.8–19.8)	0.88 (0.72–1.07)	0.91 (0.67–1.24)
>18–45	2754	49.7	19.1% (17.7–20.6)	Ref.	Ref.
>45–60	968	17.5	20.9% (18.4–23.5)	1.11 (0.93–1.34)	1.05 (0.86–1.29)
>60	647	11.7	19.3% (16.5–22.5)	1.01 (0.81–1.26)	1.00 (0.77–1.30)
Dwelling type					
Formal stand-alone house	3531	67.5	20.6% (19.3–22.0)	Ref.	Ref.
** **Informal dwelling	906	17.3	13.7% (11.6–16.1)	0.61 (0.50–0.75)	0.68 (0.55–0.84)
** **Block of flats/high-rise ** **buildings	344	6.6	20.1% (16.2–24.6)	0.97 (0.73–1.28)	0.82 (0.61–1.10)
** **Subsidized low-income ** **housing	451	8.6	22.6% (19.0–26.7)	1.13 (0.89–1.43)	1.13 (0.88–1.45)
Number of household members in the household (median; IQR; mean)	2	2–4	2.9	0.96 (0.92–0.99)	
Occupation type^a^					
** **Unemployed	2511	57.0	18.2 (16.7–19.7)	Ref.	Ref.
** **Production sector	326	7.4	24.5 (20.2–29.5)	1.55 (1.19–2.02)	1.64 (1.23–2.19)
** **Teacher, public transport, ** ** retail shop	568	12.9	22.9 (19.6–26.5)	1.38 (1.11–1.71)	1.17 (0.93–1.47)
** **Healthcare worker	160	3.6	26.9 (20.6–34.3)	1.73 (1.22–2.46)	1.56 (1.08–2.26)
** **Office work/other	304	6.9	18.8 (14.7–23.5)	1.12 (0.83–1.50)	1.06 (0.78–1.45)
** **Student	227	5.2	21.6 (16.7–27.4)	1.01 (0.83–1.22)	1.16 (0.87–1.54)
Alcohol consumption					
** **None	3406	65.6	20.3% (19.0–21.7)	Ref.	Ref.
** **Daily	146	2.8	18.5% (13.0–25.6)	0.89 (0.58–1.36)	1.29 (0.79–2.10)
** **Once or twice a week	405	7.8	17.3% (13.9–21.3)	0.82 (0.63–1.07)	0.99 (0.72–1.37)
** **Occasionally	1234	23.8	17.6% (15.6–19.8)	0.84 (0.71–0.99)	0.98 (0.81–1.18)
Smoking					
** **None	4178	80.5	20.6% (19.4–21.9)	Ref.	Ref.
** **Daily	694	13.4	11.8% (9.6–14.4)	0.52 (0.40–0.66)	0.50 (0.38–0.67)
** **Once or twice a week	158	3.0	20.3% (14.7–27.2)	0.98 (0.66–1.45)	0.87 (0.56–1.36)
** **Occasionally	161	3.1	18.6% (13.3–25.4)	0.88 (0.59–1.32)	0.92 (0.60–1.41)
Self-reported obesity					
** **No	5138	99.0	19.3% (18.3–20.4)	Ref.	Ref.
** **Yes	53	1.0	22.6% (13.3–35.8)	0.82 (0.43–1.57)	1.12 (0.57–2.21)
Multiple morbidity^a^					
** **None	4072	78.4	19.1% (17.9–20.3)	Ref.	Ref.
** **1	893	17.2	18.6% (16.2–21.3)	0.99 (0.82–1.19)	1.05 (0.85–1.29)
** **>1	226	4.4	27.4% (22.1–33.4)	1.57 (1.16–2.13)	1.66 (1.18–2.33)
District					
** **Johannesburg	1916	34.3	25.6% (23.7–27.6)	Ref.	Ref.
** **Ekurhuleni	1737	31.1	17.9% (16.2–19.8)	0.63 (0.54–0.74)	0.65 (0.55–0.78)
** **Sedibeng	378	6.8	15.9% (12.5–19.9)	0.55 (0.41–0.73)	0.51 (0.36–0.71)
** **Tshwane	946	16.9	12.7% (10.7–15.0)	0.42 (0.34–0.52)	0.41 (0.32–0.53)
** **West Rand	607	10.9	13.8% (11.3–16.8)	0.47 (0.36–0.60)	0.47 (0.35–0.62)
Month of specimen collection					
** **November 2020	1965	35.2	16.8 (15.2–18.5)	Ref.	
** **December 2020	1403	25.2	18.0 (16.5–19.7)	1.09 (0.93–1.23)	1.12 (0.94–1.34)
** **January 2021	2208	39.6	24.1 (21.9–26.4)	1.57 (1.33–1.86)	1.33 (1.10–1.61)

We determine factors associated with SARS-CoV-2 seropositivity by multivariable logistic regression adjusting for gender, age, co-morbidities, employment, self-reported obesity, district and month of specimen collection. Self-reported obesity was based on the participant reporting having been clinically diagnosed as obese. Variables significant at *p* = 0.15 in the univariable analysis were systematically added to the multivariable model assessing the model log likelihood and χ^2^. We show increased odds of SARS-CoV-2 seropositivity in females, individuals with more than one co-morbidity and individuals employed in the production sector and front-line healthcare workers. The district of residence and month of specimen collection were strongly associated with seropositivity. Unadjusted and adjusted odds ratios are presented with 95% confidence intervals (CIs) in parentheses. We used the national census classification to define dwelling types.

a Occupation and multiple morbidity restricted to individuals aged >18 years in the univariable analyses.

### Anti-SARS-CoV-2 antibody prevalence

We tested for RBD IgG in 6332 (96.1% of 6587) individuals, excluding 255 DBS with insufficient specimens for laboratory analysis. The number of samples collected varied by sampling week (Supplementary Figure S2, available as Supplementary data at *IJE* online). In five subdistricts where sampling was completed by 21 December 2020 (i.e. prior to the >10% positivity on NAAT testing), the seropositivity ranged from 9.1% to 25.0% (Table 2 and Supplementary Table S2, available as Supplementary data at *IJE* online). Of the samples obtained in January 2021, the seroprevalence was lower in the first (13.7%) rather than the third and fourth weeks of sampling (29.5 and 53.9%) (Supplementary Figure S2, available as Supplementary data at *IJE* online). Fifteen per cent (771/5131) of individuals self-reported previous NAAT testing for SARS-CoV-2 infection. The seropositivity was 46.7% [95% confidence interval (CI): 38.0–55.6; 57/122] in individuals with past self-reported NAAT-confirmed SARS-CoV-2 infection, which was 2.65-fold higher than in those who had tested negative (*n* = 114/649) on previous NAAT testing (17.6%; 95% CI: 14.9–20.8; *p* < 0001).

**Table 2:** Incidence of documented COVID-19 cases, seroprevalence of SARS-CoV-2 receptor-binding domain IgG and calculated incidence of SARS-CoV-2 infection in Gauteng Province across the districts and subdistricts

District	Subdistrict	Total population size^a^	Covid-19 cases as at 9 January 2021^b^	Documented COVID-19 cases per 1000 population	Seroprevalence (95% CI)	Calculated SARS-CoV-2 infections based on seroprevalence (95% CI)	Calculated SARS-CoV-2 infections per 1000 population (based on seroprevalence data)
Johannesburg	Johannesburg A	779 519	15 852	20.3	43.2% (37.5–49.0)	336 424 (292 495–381 778)	431.6
	Johannesburg B	435 241	17 559	40.3	29.2% (21.0–39.0)	126 945 (91 215–169 765)	291.7
	Johannesburg C	799 980	17 396	21.7	18.6% (14.4–23.7)	148 901 (115 476–189 326)	186.1
	Johannesburg D	1 396 243	27 754	19.9	23.3% (19.9–27.0)	324 944 (278 245–376 838)	232.7
	Johannesburg E	601 433	22 757	37.8	28.4% (22.0–35.8)	170 777 (132 226 –215 408)	284.0
	Johannesburg F	751 484	23 751	31.6	25.5% (20.9–30.6)	191 507 (157 369 –230 182)	254.8
	Johannesburg G	842 339	10 516	12.5	15.1% (11.1–20.2)	126 880 (93 197–169 973)	150.6
	District Total	5 606 238	135 585	24.2	25.6% (23.7–27.6)	1 436 671 (1 329 822–1 548 996)	256.3
Ekurhuleni	Ekurhuleni E1	626 517	8154	13.0	19.4% (15.9–23.3)	121 307 (99 805–146 157)	193.6
	Ekurhuleni E2	455 262	7325	16.1	18.5% (14.4–23.4)	84 073 (65 513–106 454)	184.7
	Ekurhuleni N1	708 290	14 681	20.7	18.7% (13.7–25.0)	132 318 (96 764–177 151)	186.8
	Ekurhuleni N2	697 175	19 691	28.2	17.2% (12.8– 22.8)	119 876 (89 035–158 615)	171.9
	Ekurhuleni S1	673 758	15 840	23.5	13.5% (9.3–19.1)	90 765 (62 906–128 369)	134.7
	Ekurhuleni S2	664 648	5843	8.8	18.1% (14.7–22.1)	120 117 (97 435–146 713)	180.7
	District Total	3 825 650	71 534	18.7	17.9% (16.2–19.8)	684 961 (618 664–756 686)	179.0
Sedibeng	Lesedi	127 419	1986	15.6	26.5% (16.1–40.5)	33 805 (20 478–51 618)	265.3
	Midvaal	126 285	1726	13.7	12.1% (5.9–23.2)	15 241 (7404–29 328)	120.7
	Emfuleni	830 798	14 893	17.9	14.8% (11.0–19.5)	122 627 (91 508–162 010)	147.6
	District Total	1 084 503	18 605	17.2	15.9% (12.5–19.9)	172 143 (135 833–215 951)	158.7
City of Tshwane	Tshwane 1	1 032 885	19 897	19.3	11.9% (8.5–16.5)	123 152 (87 905–169 989)	119.2
	Tshwane 2	436 950	6383	14.6	10.4% (7.0–15.2)	45 474 (30 645–66 309)	104.1
	Tshwane 3	730 788	27 335	37.4	9.4% (5.8–14.8)	68 780 (42 717–108 243)	94.1
	Tshwane 4	482 448	12 428	25.8	5.5% (1.8–15.6)	26 315 (8537–75 242)	54.5
	Tshwane 5	119 190	1569	13.2	10.9% (4.6–23.6)	12 955 (5479–28 112)	108.7
	Tshwane 6	768 446	15 567	20.3	20.8% (15.1–27.9)	159 677 (115 950–214 474)	207.8
	Tshwane 7	138 928	1702	12.3	25.0% (14.0–40.5)	34 732 (19 464–56 329)	250.0
	District Total	3 709 635	84 881	22.9	12.7% (10.7–15.0)	470 567 (397 335–555 035)	126.8
West Rand	Mogale City	435 254	10 448	24.0	16.5% (12.4–21.5)	71 689 (53 994–93 756)	164.7
	Rand West City	300 960	5559	18.5	9.1% (5.6–14.3)	27 360 (16 984–43 112)	90.9
	Merafong City	213 874	3724	17.4	14.8% (10.7– 15.0)	31 595 (22 908–32 000)	147.7
	District Total	950 088	19 731	20.8	13.8% (11.3–16.8)	131 478 (107 480–159 819)	138.4
Provincial total	Gauteng Province	15 176 113	330 336	21.8	19.1% (18.1–20.1)	2 897 120 (2 743 907–3 056 867)	190.9

We estimate the seroprevalence of SARS-CoV-2 receptor-binding domain (RBD) IgG in 5584 individuals sampled across 26 subdistricts in Gauteng, South Africa from 4 November 2020 to 22 January 2021. The threshold indicative of seropositivity for SARS-CoV-2 RBD was selected as IgG 26 BAU/mL, based on the highest value of RBD IgG in samples from the pre-COVID-19 era. Seroprevalence was calculated as the number of individuals who were seropositive divided by the total number of individuals sampled. We present the overall provincial seroprevalence and the district- and subdistrict-specific seroprevalence 95% CIs are given in parentheses. The seroprevalence for Gauteng was 19.1%, ranging 12.7–25.6% across districts and 9.1–43.2% across subdistricts. We show that the calculated number of SARS-CoV-2 infections was 8-fold higher than number of documented COVID-19 cases, ranging from 1.1- to 20-fold higher across subdistricts.

a Population estimates obtained from the STATS-SA provincial mid-year population estimates.

b COVID-19 cases obtained from the National Institute for Communicable Diseases weekly COVID-19 report and National Department of Health daily statistics.

The seroprevalence was 22.2% (95% CI: 19.4–25.3%) in 748 individuals who had inadequate documentation of place of residence, who were excluded from the district and subdistrict analyses. The seroprevalence was 19.1% (95% CI: 18.1–20.1%) in individuals with an identifiable residential address (Table 2). The seroprevalence was similar across age groups <5 (18.0%, 95% CI: 14.1–22.7), 5–18 (17.2%, 95% CI: 14.8–19.8), >18–45 (19.1%, 95% CI: 17.7–20.6), >45–60 (20.9%, 95% CI: 18.4–23.5) and >60 (19.3%, 95% CI: 16.5–22.5) years (Table 1).

### SARS-CoV-2 RBD IgG seroprevalence by geographic area

**Figure 2:**
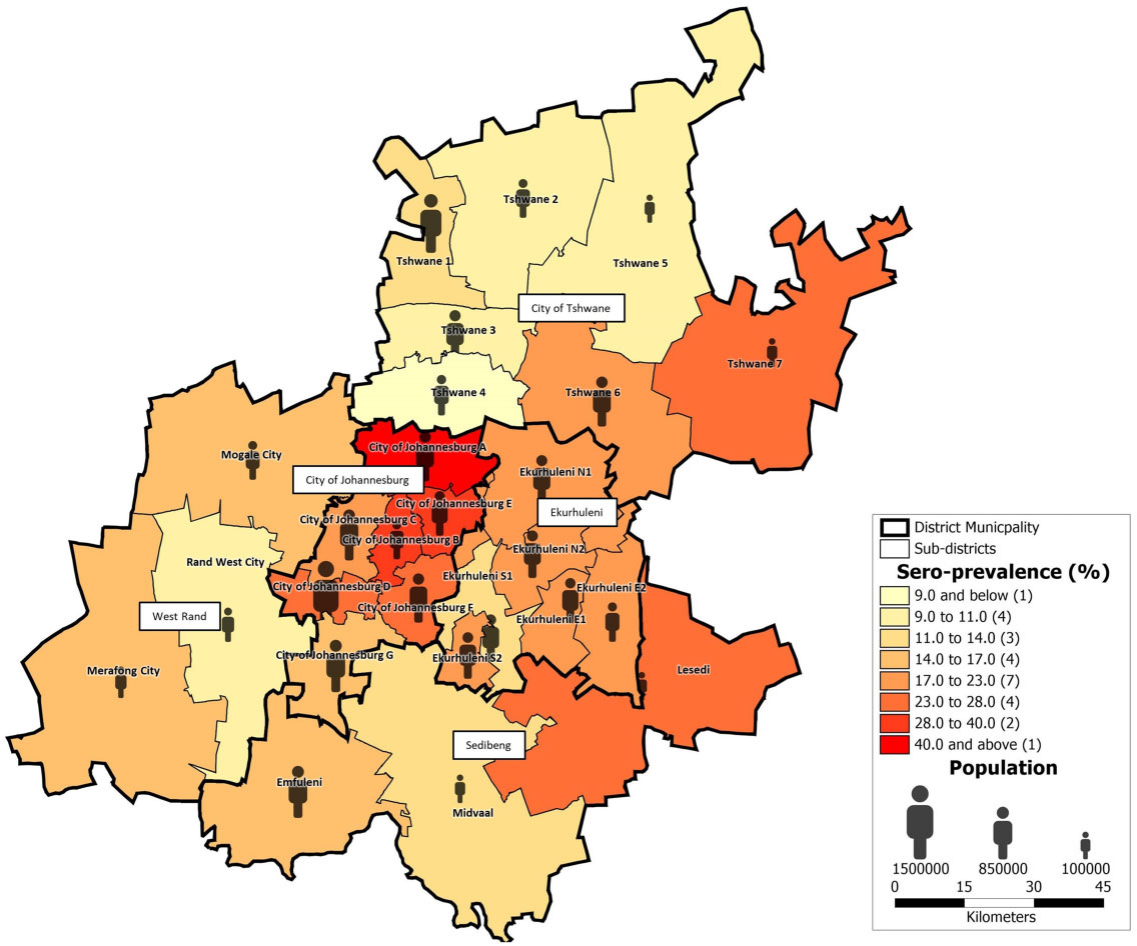
SARS-CoV-2 seroprevalence by subdistrict. SARS-COV-2 seroprevalence is presented by subdistrict showing heterogeneity across the districts and subdistricts. Seroprevalence is presented in relation to the population and geographic size of each region. City of Johannesburg, the smallest in geographic size but with the largest population size, has the highest seroprevalence.

The seroprevalence varied across districts. The highest was in Johannesburg (25.6%, 95% CI: 23.7–27.6), where 10.4% of residences were informal dwellings and the population density is 3400 per km^2^, and the lowest was in Tshwane (12.7%; 95% CI: 10.7–15.0), where 23.3% of dwellings were informal structures and population density is 460 per km^2^ (Table 2, Figure 2 and Supplementary Table S2, available as Supplementary data at *IJE* online). The seroprevalence in the other three districts was 13.8% in West Rand (population density 200 per km^2^), 15.9% in Sedibeng (population density 198 per km^2^) and 17.9% in Ekurhuleni (population density 1600 per km^2^) (Table 2).

**Figure 3:**
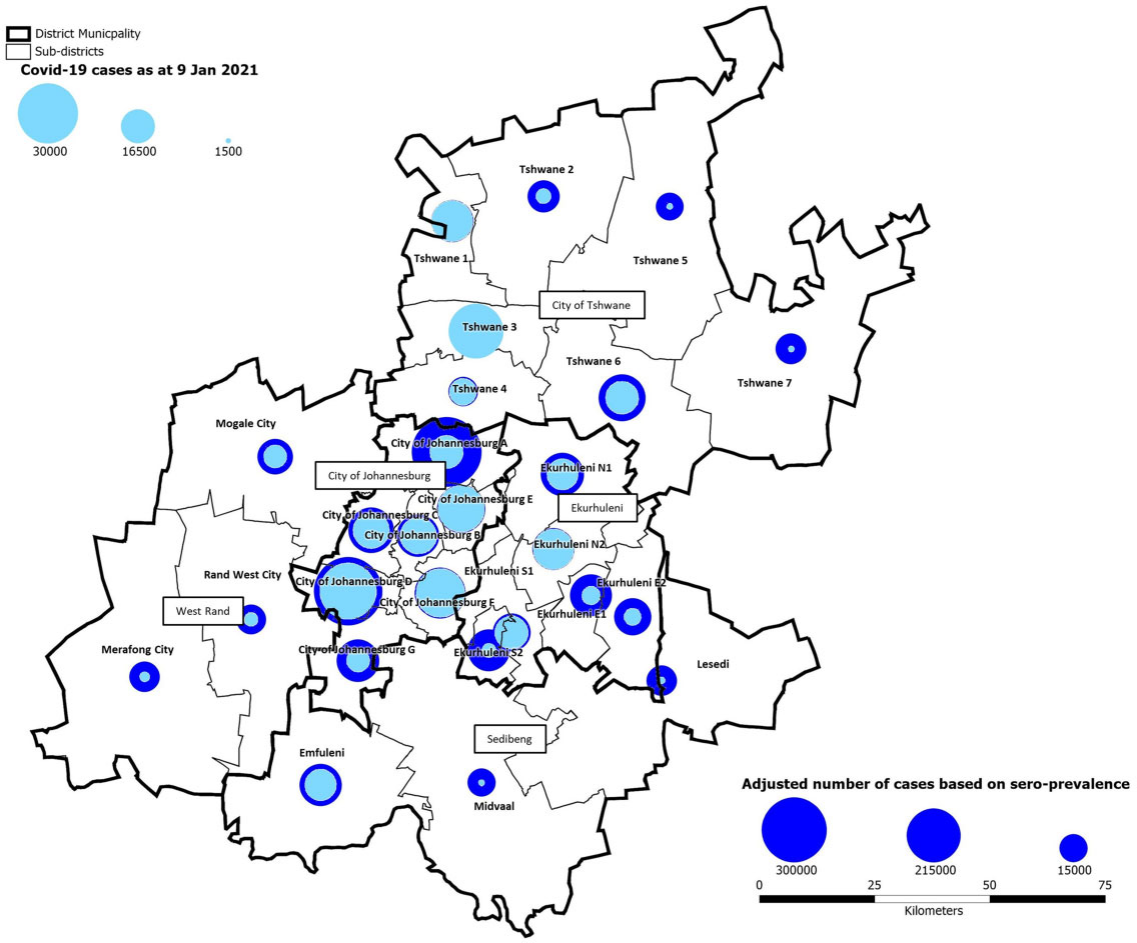
Subdistrict reported COVID-19 cases (through to 9 January 2021) compared with calculated SARS-CoV-2 infections. The adjusted number of infections was calculated by applying the seroprevalence to the population size at provincial, district and subdistrict levels. Across all subdistricts except for two districts, the documented COVID-19 cases significantly underestimated the population-level SARS-CoV-2 infections.

There was also heterogeneity in seroprevalence across subdistricts ranging from 5.5% (95% CI: 1.8–15.6) in Tshwane region-4 to 43.2% (95% CI: 37.5–49.0) in Johannesburg region-A (Table 2 and Figure 2). In Johannesburg, the seroprevalence across subdistricts varied from 15.1% in region-G to 43.2% in region-A. Similarly, the seroprevalence varied in Sedibeng (12.1–26.5%) and Tshwane (5.5–25.0%) across subdistricts. Across all districts, the difference between reported COVID-19 cases and infections estimated from seroprevalence was >5-fold and >15-fold in four subdistricts (Table 2 and Figure 3).

### Factors associated with anti-SARS-CoV-2 antibody seropositivity

In adjusted multivariable logistic regression, characteristics associated with higher seropositivity included female gender (adjusted odds ratio (aOR): 1.24; 95% CI: 1.06–1.45), more than one underlying chronic illness (aOR: 1.66; 95% CI: 1.18–2.33), employment as a healthcare worker (aOR: 1.56; 95% CI: 1.08–2.26) or in production industry (aOR: 1.64; 95% CI: 1.23–2.19) compared with unemployed individuals. Compared with individuals sampled in 2020, individuals sampled in January 2021 had 1.33 (95% CI: 1.10–1.61) higher odds of being seropositive. Seropositivity was lower in individuals who lived in an informal compared with those in formal stand-alone dwellings (aOR: 0.68; 95% CI: 0.55–0.84) and in daily smokers compared with non-smokers (aOR: 0.50; 95% CI: 0.38–0.67) (Table 1). Adjusting for the time period of sample collection, the odds of seropositivity were lower in Ekurhuleni (aOR: 0.65), Sedibeng (aOR: 0.51), Tshwane (aOR: 0.41) and West Rand (aOR: 0.47) than in Johannesburg (Table 1).

### Calculated SARS-CoV-2 infections

Overall, we estimated that there were 2 897 120 (95% CI: 2 743 907–3 056 867) SARS-CoV-2 infections using the seroprevalence data, which was 7.8-fold higher than the number of COVID-19 cases (*n* = 330 336) reported in Gauteng until 9 January 2021 (Table 2). Further, the calculated number of SARS-CoV-2 infections (per 1000 population) was 190.9 for Gauteng, highest in Johannesburg (256.3), lowest in Tshwane (126.8) and ranging from 138.4 to 179.0 in other districts. In contrast, the number (per 1000 population) of documented COVID-19 was 21.8 in Gauteng and similar in Johannesburg (24.2) compared with Tshwane (22.9) and ranging from 17.2 to 20.8 in other districts as of 9 January 2021. The calculated number of SARS-CoV-2 infections was 7.8-fold higher than documented COVID-19 cases across the Province, varying 4.5- to 9.6-fold across the districts.

The calculated number (per 1000 population) of SARS-CoV-2 cases also differed between subdistricts, being highest in Johannesburg region-A (431.6) and lowest in Tshwane region-4 (54.5) (Table 2). The number of documented COVID-19 cases (per 1000 population) in Johannesburg subdistricts ranged from 12.5 to 40.3, which was 6.2- to 20.2-fold lower than the calculated number of SARS-CoV-2 infections (Table 2).

### COVID-19 CFR and SARS-CoV-2 IFR

As of 24 January 2021, there were 8198 recorded COVID-19 deaths in Gauteng, for an overall COVID-19 case-fatality risk of 2.1% (8198/3884.620), ranging from 1.7% in Johannesburg to 2.8% in West Rand. The rate (per million population) of documented COVID-19 deaths for Gauteng was 540 (range 452–669 in the districts) (Table 3). The excess mortality from natural causes of death in individuals >1 year old was 21 582 in Gauteng from 25 April 2020 until 24 January 2021.^16^ Triangulating with the calculated number of SARS-CoV-2 infections, the mortality ratio was 0.28% (8198/2 897 120) based on recorded COVID-19 deaths and 0.67% (19 424/2 897 120) based on the assumption that 90% of the excess mortality was due to COVID-19. The district estimates for mortality risk triangulated with calculated SARS-CoV-2 infection ranged between 0.18% and 0.48% for reported COVID-19 deaths and 0.47% and 0.99% based on COVID-19 assumed deaths from excess-mortality estimates. The documented provincial mortality rate (per million population) assuming that 90% of excess natural deaths were due to COVID-19 was 1280, ranging from 1197 in Johannesburg to 1379 in Ekurhuleni (Table 3).

**Table 3:** Adjusted SARS-CoV-2-infection fatality rates based on reported COVID-19 deaths and excess mortality

	Population size	Documented COVID-19 cases	Documented COVID-19 deaths	Crude mortality rate per million population-based on documented COVID-19 deaths	COVID-19 CFR based on documented COVID-19 deaths	Calculated SARS-CoV-2 infections (95% CI)	Calculated IFR based on community-based seroprevalence (95% CI)	Total excess mortality^20^	Number of COVID-19 deaths, assuming 90% excess mortality due to COVID-19	Calculated IFR assuming 90% excess mortality is due to COVID-19	Calculated SARS-CoV-2 mortality rate per million population
Johannesburg	5 606 238	154 137	2536	452	1.6%	1 436 671 (1 329 822–1 548 996)	0.18% (0.16–0.19)	7454	6709	0.47% (0.43–0.50)	1197
Ekurhuleni	3 825 650	83 271	1915	501	2.3%	684 961 (618 664–756 686)	0.28% (0.25–0.31)	5862	5276	0.77% (0.70–0.85)	1379
Sedibeng	1 084 503	21 954	588	542	2.7%	172 143 (135 833–215 951)	0.34% (0.27–0.43)	^a^No data			
Tshwane	3 709 635	103 011	2478	668	2.4%	470 567 (397 335–555 035)	0.53% (0.45–0.62)	5186	4667	0.99% (0.84–1.17)	1258
West Rand	950 088	22 628	636	669	2.8%	131 478 (107 480–159 819)	0.48% (0.40–0.59)	^a^No data			
Gauteng Province	15 176 113	388 620	8198	540	2.1%	2 897 120 (2 743 907–3 056 867)	0.28% (0.27–0.30)	21 582	19 424	0.67% (0.64–0.71)	1280

We compute the provincial and district case-fatality ratio (CFR) by dividing the documented number of deaths by the total documented COVID-19 cases and the infection fatality ratio (IFR) by dividing the documented deaths by the calculated number of SARS-CoV-2 infections. Sensitivity analysis was performed by calculating the IFR assuming that 90% of the excess mortality since the onset of the pandemic was due to COVID-19. The estimated numbers of deaths are used to estimate the excess natural deaths experienced in areas that have increased above the upper prediction level. We show that CFR is overestimated using documented COVID-19 cases due to the underestimation of SARS-CoV-2 infections. The calculated provincial IFR was 0.67 compared with the 2.1% CFR based on documented COVID-19 deaths and cases.

a No data available on excess mortality within these districts.

## Discussion

In Gauteng, the most densely populated province in South Africa, where 25% of the population of the country live, the seroprevalence for SARS-CoV-2 infection was 19.1% after the first COVID-19 wave through to the early phase of the resurgence in mid-January 2021. The calculated number of SARS-CoV-2 infections (2 897 120) in Gauteng was at least 8-fold higher than the documented number of COVID-19 cases (*n* = 330 336) until 9 January 2021. Triangulating the calculated number of SARS-CoV-2 infections from the seroprevalence survey with reported or excess mortality calculated COVID-19 deaths yielded mortality-risk estimates of 0.28% for reported COVID-19 deaths and 0.67% based on the assumption that 90% of the excess mortality was due to COVID-19. The possible waning of SARS-CoV-2-infection-induced IgG, which reportedly occurs within 50–90 days of previous asymptomatic infection or mild COVID-19,^20^^,^^23^^,^^24^ could have contributed to 53.3% of individuals in our survey who self-reported past NAAT-confirmed COVID-19 being seronegative. The implications of the probable under-ascertainment of past infections could have resulted in a 2-fold underestimate of the calculated number of SARS-CoV-2 infections in Gauteng at the time of the survey.

A meta-analysis of 61 studies, including 24 with random sampling from the general population of which 5 were performed in areas with high COVID-19 cases or deaths, yielded seroprevalence estimates of 0.02–53.4% and calculated infection fatality risks ranging from 0% to 1.63%.^25^ In the single African country (Kenya) included in the meta-analysis, the crude seroprevalence was 5.6% in blood-donor samples with the inferred infection-mortality risk of 0.00% based on 341 documented cumulative deaths having occurred among the calculated 2 783 453 SARS-CoV-2 infections estimated to have occurred by 31 July 2020.^25^^,^^26^ The mortality risk in our study varied by 2.39-fold when calculated using reported COVID-19 deaths (0.28%) compared with COVID-19 calculated deaths using excess-mortality data (0.67%), both estimates being in the mid or lower range reported in the meta-analysis.^25^ The mortality risk in Gauteng could, however, be 2-fold lower if adjusted for the possibility of us having underestimated the number of SARS-CoV-2 infections due to the waning of IgG in previously infected individuals. Nevertheless, the high force of SARS-CoV-2 infection prior to the peak of the COVID-19 resurgence resulted in an infection rate (per million population) of 540 for recorded COVID-19 deaths and 1280 based on 90% of the non-accidental excess-mortality deaths being attributed to COVID-19 in Gauteng. Both these mortality rates are higher than the global reported COVID-19 mortality rates of 426 per million.^27^ The mortality rate (per million population) calculated from excess mortality attributable COVID-19 deaths in Gauteng (1280) is 1.39-fold greater than the corresponding overall reported mortality rate in South Africa (675) as of 22 January 2021.^27^ The cumulative recorded COVID-19 deaths (per million people) from mainly high-income countries ranged from 467 to 1783 as of 22 January 2021,^27^ although differences in the completeness of the documentation of COVID-19 deaths limits any head-to-head comparisons between settings. Also, we are unaware of the availability of excess-mortality data to estimate COVID-19-attributable deaths from any other sub-Saharan Africa at this stage, to make further intercontinental comparisons.^28^^,^^29^

Corroborating our findings of a high force of SARS-CoV-2 infection in South Africa during the course of the first COVID-19 wave are other convenience-sampling serosurveys undertaken soon after the first wave had subsided to its trough. In the Cape Metro (Western Cape Province, South Africa), convenience testing of residual blood samples obtained from people living with HIV and women attending antenatal clinics ∼2 weeks after the peak of the first wave reported a seroprevalence of 31–46% using the Roche Elecsys anti-SARS-CoV-2 assay.^30^ Similarly, the seroprevalence using an N-protein IgG assay was 30% among volunteers who had enrolled in a phase IIb COVID-19 vaccine-efficacy trial in South Africa (the majority in Gauteng) in which enrolment commenced from mid-July 2020, i.e. soon after the first wave had subsided.^31^ Furthermore, a serosurvey of samples from blood donors in four provinces undertaken during the peak of the second wave between 7 and 25 January 2021 reported a SARS-CoV-2 IgG seropositivity of 31.8–62.5% using the Elecsys Anti-SARS-CoV-2 immunoassay.^32^ Together, these data corroborate our findings of a high force of SARS-CoV-2 infection having taken place in South Africa following the first and second COVID-19 waves.

Our survey also illustrates the heterogeneity of SARS-CoV-2 infection between districts, and even within subdistricts in a district. The force of infection in our survey was greatest in Johannesburg region-A (43% seropositivity), which includes both affluent and high-density areas. In contrast, seropositivity was only 5.5% in Tshwane region-4, one of the most affluent areas in Gauteng situated in a nature-reserve area. Delineating subdistrict differences in the previous number of SARS-CoV-2 infections could assist in tailoring future public health initiatives aimed at mitigating the consequences of further resurgences of COVID-19. This could include selecting which subdistricts to prioritize in the roll-out of COVID-19 vaccines in countries such as South Africa, and almost all other African countries, which are faced with limited immediate access to COVID-19 vaccines.

The seroprevalence observed in our study is similar to that estimated across four population-based serosurveys after the first wave in South East Asia (19.6%; 95% CI: 5.5–33.6) as reported in a meta-analysis.^9^ Elsewhere in population-based and convenience-sampling serosurveys, also mainly after the first COVID-19 waves, the reported seroprevalence was 16.3% (95% CI: 0.0–33.7) in Africa (*n* = 2), 13.4% (95% CI: 8.8–18.0) in the Eastern Mediterranean (*n* = 19), 6.8% (95% CI: 5.0–8.5%) in the Americas (*n* = 120) and 4.7% (95% CI: 3.6–5.9) in Europe (*n* = 194).^9^ Previous studies have reported the ratio of serologically detected infections relative to virologically confirmed cases ranging from 6.9 to 92.^9^^,^^13^^,^^33^^,^^34^ In our survey, the ratio of seropositivity compared with reported COVID-19 cases was >10 in eight subdistricts and >20 in three subdistricts, which is likely to be an underestimate due to the possible waning of IgG in previously infected individuals. Considering that South Africa has the highest testing rate for active SARS-CoV-2 infection in Africa,^2^^,^^4^ this alludes to an even greater underestimation of COVID-19 elsewhere in Africa. In the Zambian population-based survey, the ratio of seropositivity to reported COVID-19 cases was 92:1 overall and up to 1012:1 in some districts.^13^ Notably, differences in the timing of the serosurvey sampling in relation to the trajectory of the COVID-19 pandemic, which differs between countries, limits the validity of such head-to-head comparisons between studies.

Interestingly, despite the prolonged school closures in South Africa for the entire period of the first COVID-19 wave (from 26 March 2020 until 31 May 2020), the seropositivity in children <5 (18.0%) and 5–18 (17.2%) years old was similar compared with that in adults (19.1–20.9%). We did, however, observe that females had 1.24-fold higher odds of being seropositive, in contrast to no gender-associated differences observed in a meta-analysis^9^^,^^10^ or the Zambian serosurvey.^13^ We also observed that employment as a healthcare worker (aOR: 1.56) and in the production sector (aOR: 1.64) was associated with higher seropositivity than being unemployed. Reasons for this could include the increased mobility of such categories of workers having to travel to their place of employment (often by public transport in South Africa) and their greater likelihood of working indoors and in settings of high SARS-CoV-2 exposure such as health facilities. The lower prevalence of seropositivity in people living in informal dwellings (aOR: 0.68) could possibly be due to fewer people per household in such structures and residents of such dwellings being more likely to be unemployed. Although a meta-analysis associated smoking with greater risk of developing severe COVID-19,^35^ surprisingly, we observed that individuals who self-reported being daily smokers had 0.50-fold lower odds of being seropositive than non-smokers. The reason for regular smoking being negatively associated with seropositivity is unclear, although lower antibody levels following SARS-CoV-2 infection have been reported in smokers.^36^ It is possible that an attenuated humoral immune response in regular smokers could lead to more rapid decline in IgG and consequently the under-ascertainment of past SARS-CoV-2 infection. There is no information to our knowledge that provides a biological basis as to why regular smokers might have a reduced risk of SARS-CoV-2 infection.

Limitations of our study include that the sampled population age-group structure was different to the general population; the median age using 2020 provincial mid-year population estimates was 28 years whereas the median age of the participants in our survey was 34 years. Also, we surveyed a higher percentage of females (60%) compared with the mid-year estimates for the general population in 2020 (50%), which could have led to an overestimate in the seroprevalence, as female gender was independently associated with higher seropositivity. Furthermore, we used an in-house RBD IgG assay, which was however validated for high sensitivity and specificity using acute phase convalescent sera from COVID-19 cases and was benchmarked to the World Health Organization (WHO)-recommended standardized reference sera. Nevertheless, antibody tests have limitations,^37^ including the presence of pre-existing SARS-CoV-2 RBD IgG due to other seasonal coronaviruses. The waning of antibodies was suggested by 53% of individuals with self-reported past NAAT-confirmed SARS-CoV-2 infection testing as seronegative.

## Supplementary data

Supplementary data are available at *IJE* online.

## Ethics approval

The University of the Witwatersrand Human Research Ethics Committee granted a waiver for formal approval of the survey, which was deemed to be part of public health good and surveillance to manage the COVID-19 pandemic. Electronic signed informed consent was administered to individuals >15 years old, parental consent obtained for children <12 years of age, and assent and parental consent for adolescents 12–15 years old.

## Funding

This work was supported by the Bill and Melinda Gates Foundation (Grant number 023514).

## Data availability

De-identified individual-level data and data sets generated during the current study are available for researchers who provide a methodologically sound proposal. If approved, the requestor must sign a data-use agreement. Additionally, the study protocol is available on request. All requests must be addressed to the corresponding author. Data will be available 3 months after publication of this manuscript.

## Supplementary Material

dyab217_Supplementary_Data
