# Supplementary material for: Estimated SARS-CoV-2 infection rate and fatality risk in Gauteng Province, South Africa: a population-based seroepidemiological survey
Source: Int J Epidemiol. 2021 Oct 30;51(2):404–17. doi: 10.1093/ije/dyab217 (PMC8689871; doi:10.1093/ije/dyab217)
Supplement: dyab217_Supplementary_Data [file dyab217_supplementary_data.pdf]

Supplementary material:

# Estimated SARS-CoV-2 infection rate and fatality risk in Gauteng Province, South Africa: a population based sero-epidemiological survey.

## 1.1 Epidemiology of COVID-19 in Gauteng and Government responses

The first confirmed COVID-19 case in Gauteng was identified on March 7, 2020. South Africa's Government embarked on a highly restrictive lockdown from March 23, 2020, with almost complete shutdown of all economic and public activities until May 31, 2020, following which restrictions were gradually relaxed paradoxically as the first COVID-19 wave gained momentum (Supplementary Fig. S1). The effective reproductive rate for COVID-19 was estimated at 1·5 during the initial highly restrictive lockdown, indicating ongoing community transmission of SARS-CoV-2[1]. Despite availability of medical laboratory infrastructure in South Africa, testing for SARS-CoV-2 using NAAT was constrained by challenges in accessing testing reagents, resulting in testing for SARS-CoV-2 infection being mainly selective of symptomatic or highly suspicious cases of COVID-19[2]. The selective testing was partly manifest by high average test positivity rates of 29·7% and 34·7% during the peak periods, defined as when the rolling 7-day average for NAAT positivity rates was >10%. The timing of the peak of the first wave was from mid-June to early-September, 2020 and the second peak was from mid-November, 2020 to end-January, 2021[3].

**Supplementary Table S1: Literature review of sero-surveys conducted in Africa through to April 30, 2021.**

| Author and publication status | Country & setting | Study population | Design | Sample size | Participant age (years)<br><br>Median (IQR) | Specimen collection dates | Survey in relation to waves | Antibodies tested | Assay used | Sensitivity / specificity | Seroprevalence (%) |
|---|---|---|---|---|---|---|---|---|---|---|---|
| Sykes et al·, (2021)· Preprint | South Africa· 4 provinces | Blood donors – blood transfusion centres | Cross-sectional | 4 858 | 15-69 | January 7-25, 2021 | 2nd wave peak and soon after | IgG | Elecsys Anti-SARS-CoV-2 immunoassay | 99/100 | 31·8 to 62·5 |
| Uyoga et al·, (2021)· Published | Kenya· National | Blood donors – 4 regional blood transfusion centres | Cross-sectional | 3 174 | 15-66 | April 30-June 16, 2020 | 1st wave surge | IgG | ELISA - spike IgG | 93/99 | 5·6 |
| Alemu et al·, (2020)· Published | Ethiopia· Addis Ababa | Random sample of adult community members | Cross-sectional | 301 | 30 (19-41) | April 23-28, 2020 | Pre-1st wave surge | IgG, IgM | Lateral flow immunoassay | 88/100 | 7·6 |
| Quashie et al·, (2021)· Preprint | Ghana· Selected regions | Convenience sample of adults at markets, lorry stations, malls, Covid testing centres, hospitals | Repeated cross-sectional | 2 729 | Mode: 21-40 | *August 2020; October 2020; December 2020; February 2021 | Start: post-1st wave<br><br>End: peak 2nd wave | IgG IgM | Lateral flow immunoassay | 98/64 | Round 1: IgG 13·0; IgM 1·6<br><br>Round 2: IgG 15·2; IgM 1·3<br><br>Round 3: IgG 9·7; IgM 2·3<br><br>Round 4: IgG 22·8, IgM 2·2 |
| Kempen et al·, (2020)· Published | Ethiopia· Addis Ababa | Convenience sample of laboratory users | Cross-sectional | 99 | >14 | May 18-21, 2020 | Pre-1st wave surge | IgG | Abbott IgG | 100/100 | 3·0 |
| Majiya et al·, (2020)· Preprint | Nigeria· Niger State | Random sample of adult community members | Cross-sectional | 185 | >30 | June 26-30, 2020 | 1st wave peak | IgG, IgM | Lateral flow immunoassay | 99/98 | IgG: 25·4<br>IgM: 2·2 |

| | | | | | | | | | | | |
|---|---|---|---|---|---|---|---|---|---|---|---|
| Mulenga et al·, (2021)· Published | Zambia· Six districts | Population-based representative sample of 6 districts | Cross-sectional | 3 742 | 18 (8–32) | July 4-27, 2020 | 1st wave surge and peak | IgG | ELISA (spike) | 90/N/A | Pooled: 10·6<br><br>ELISA only: 2·1 |
| Wiens et al·, (2021)· Preprint | South Sudan· Juba | Population-based representative sample of Juba | Cross-sectional | 2 214 | 1-84 | August 10-Sep 11, 2020 | Post-1st wave | IgG | ELISA - spike RBD | 100/64 | 22·3 |
| Hsiao et al·, (2021)· Unpublished report | South Africa· Cape Town | Residual blood of patients at primary care clinics | Cross-sectional | 2 791 | <20->50 | July 15-August 7, 2020 | 1st wave peak and soon after | IgG, IgM | Roche Elecsys anti-SARS-CoV-2 assay | 91/100 | 40·0 |
| Halatoko et al·, (2020)· Published | Togo· Lome | Convenience sample of high-risk adults | Cross-sectional | 955 | 36 (32-43) | April 23, 2020, to May 8, 2020 | Months before 1st wave | IgG, IgM | Rapid Test | 73/85 | Pooled 1·6<br><br>IgG or IgM: 0·9 |
| Mukwege et al·, (2021)· Published | Democratic Republic of Congo· Bukavu | Health workers at 1 hospital | Cross-sectional | 359 | mean 43,1 (SD 11,3) | July 2-August 19, 2020 | Post-1st wave peak | IgG, IgM | Lateral flow immunoassay· ELISA to confirm | 62/99 | 41·2 |
| Mostafa et al·, (2021)· Published | Egypt· Cairo | Health workers at 12 health facilities | Prospective cohort | 4 040 | 32 (27-42) | April 22-June 10, 2020 | 1st wave surge and peak | IgG, IgM | Lateral flow immunochromatographic assay | 83/100 | IgG: 0·4,IgM: 1·0 |
| Kassem et al·, (2020)· Published | Egypt· Cairo | Health workers at 1 hospital | Cross-sectional | 74 | 32 (x-x) | June 1-14, 2020 | 1st wave peak | IgG, IgM | Rapid Serology Test | 93/98 | IgG + IgM: 4·0<br>IgG or IgM: 12·2 |
| Rusakaniko et al·, (2021)· Published | Zimbabwe· Bulawayo | Health workers at 24 health facilities | Cross-sectional | 635 | 40 (32–52) | June 9-22, 2020 | Pre-1st wave surge | IgG, IgM | Chromatographic immunoassay (spike & nucleocapsid) | 98/88 | IgG or IgM: 8·9 |
| Etyang et al·, (2021)· Preprint | Kenya· 3 counties | Health workers at 5 hospitals | Cross-sectional | 684 | 35 (24-46) | July 3-December 4, 2020 | 1st wave peak to post-2nd wave peak | IgG | ELISA | 93/99 | 19·7 |
| Chibwana et al·, (2020)· Preprint | Malawi· Blantyre | Health workers at 1 hospital | Cross-sectional | 500 | 31 (20-64) | May 22-June 19, 2020 | Pre-1st wave surge | IgG | ELISA (spike & nucleo) | 88/93 | 16·8 |

| | | | | | | | | | | | |
|---|---|---|---|---|---|---|---|---|---|---|---|
| Abdelmoniem et al·, (2021)· Published | Egypt· Cairo | Health workers at 1 hospital | Cross-sectional | 203 | 31·9 (25,3-38,5) | June 1-14, 2020 | 1st wave peak | IgG, IgM | Rapid test, quali· lateral flow immunochromatographic assay | 93/98 | IgG + IgM: 16·7 IgG or IgM: 18·2 |
| Olayanju et al·, (2021)· Published | Nigeria· Ibadan | Health workers at 1 hospital | Cross-sectional | 133 | 20-60 | N/A | Pre-1st wave surge | IgG | ELISA | N/A | 45·1 |
| Kammon et al·, (2020)· Preprint | Libya· Alzintan | Adult patients and health workers at 2 hospitals and 1 clinic | Cross-sectional | 219 | Adults | April 2-May 18, 2020 | Pre-1st wave (survey ended before 20 cases) | Combined IgG+ IgM | Lateral flow immunochromatographic assay | 86/99 | 2·7 |
| Asuquo et al·, (2020)· Preprint | Nigeria· Calabar | Adult patients and health workers at 1 hospital | Cross-sectional | 66 | > 18 | June 17-25, 2020 | 1st wave peak | IgG, IgM | COVID-19 IgG/IgM Rapid Test + another test kit (not well described) | N/A | IgG: 25·8 IgG + IgM: 7·6 |
| Shaw et al·, (2021)· Published | South Africa· Cape Town | Convenience sample of workers at 1 shopping & tourist complex | Cross-sectional | 405 | 38 (18–69) | August 17-September 4, 2020 | Post-1st wave peak | IgG | Abbott SARS-CoV-2 IgG assay | 87/99 | 23·7 |
| Kagucia | Kenya· 3 counties | Convenience sample of truck drivers and assistants | Cross-sectional | 830 | 40 (34-48) | Sep 30-October 23, 2020 | 2nd wave surge | IgG | ELISA (spike) | 93/99 | 39·6, |

*The four rounds were not done in all sites or in the same sites.

We identified 22 African seroprevalence surveys through a literature search. We searched PubMed for African seroprevalence studies published during 1 January 2020 to 25 April 2021, using the key words “COVID-19”, “SARS-COV-2”, “seroprevalence”, “antibody survey”, “sero-epidemiolog*”, and “Africa”. We also searched the pre-print server MedRxiv. We identified 17 studies. We further used the Google search engine to identify other pre-prints and unpublished reports and reviewed the bibliographies of our identified studies and recent systematic reviews. This increased the identified studies to 22.

**Supplementary Table S2: survey sample size per sub-district and the accompanying seroprevalence estimates**

| District | Sub-district | Total population size | % of informal dwellings sampled | Target sample size | Sampled size | Achieved as % of target | Survey start date | Survey end date |
|---|---|---|---|---|---|---|---|---|
| Johannesburg | Johannesburg A | 779 519 | 5·8% | 287 | 285 | 99·2% | 07-Dec-20 | 22-Jan-21 |
| | Johannesburg B | 435 241 | 0·0% | 142 | 96 | 67·6% | 08-Dec-20 | 21-Jan-21 |
| | Johannesburg C | 799 980 | 7·3% | 301 | 274 | 91·1% | 16-Nov-20 | 14-Jan-21 |
| | Johannesburg D | 1 396 243 | 12·4% | 508 | 550 | 108·3% | 04-Nov-20 | 21-Jan-21 |
| | Johannesburg E | 601 433 | 15·8% | 174 | 162 | 93·2% | 16-Nov-20 | 21-Jan-21 |
| | Johannesburg F | 751 484 | 9·7% | 217 | 310 | 142·8% | 17-Dec-20 | 20-Jan-21 |
| | Johannesburg G | 842 339 | 16·4% | 319 | 239 | 74·8% | 05-Nov-20 | 18-Jan-21 |
| | Johannesburg District | 5 606 238 | 10·4% | 1 948 | 1 916 | 98·4% | 04-Nov-20 | 21-Jan-21 |
| Ekurhuleni | Ekurhuleni E1 | 626 517 | 15·8% | 283 | 439 | 155·4% | 09-Nov-20 | 18-Jan-21 |
| | Ekurhuleni E2 | 455 262 | 25·5% | 201 | 287 | 142·4% | 10-Nov-20 | 18-Jan-21 |
| | Ekurhuleni N1 | 708 290 | 17·8% | 276 | 182 | 66·0% | 09-Nov-20 | 21-Jan-21 |
| | Ekurhuleni N2 | 697 175 | 21·4% | 268 | 221 | 82·5% | 12-Nov-20 | 21-Dec-20 |
| | Ekurhuleni S1 | 673 758 | 29·9% | 257 | 193 | 75·1% | 07-Dec-20 | 22-Jan-21 |
| | Ekurhuleni S2 | 664 648 | 16·3% | 299 | 415 | 138·6% | 25-Nov-20 | 22-Jan-21 |
| | Ekhuruleni District | 3 825 650 | 20·0% | 1 584 | 1 737 | 109·7% | 09-Nov-20 | 22-Jan-21 |
| Sedibeng | Emfuleni | 830 798 | 18·4% | 369 | 271 | 73·4% | 12-Nov-20 | 21-Jan-21 |
| | Lesedi | 127 419 | 33·3% | 56 | 49 | 87·0% | 23-Nov-20 | 16-Jan-21 |

| | | | | | | | | |
|---|---|---|---|---|---|---|---|---|
| | Midvaal | 126 285 | 27·1% | 51 | 58 | 113·4% | 10-Dec-20 | 19-Jan-21 |
| | Sedibeng District | 1 084 503 | 21·8% | 477 | 378 | 79·3% | 12-Nov-20 | 21-Jan-21 |
| Tshwane | Region 1 | 1 032 885 | 25·5% | 468 | 260 | 55·6% | 10-Nov-20 | 14-Jan-21 |
| | Region 2 | 436 950 | 24·5% | 176 | 221 | 125·3% | 06-Nov-20 | 17-Jan-21 |
| | Region 3 | 730 788 | 20·4% | 262 | 170 | 65·0% | 20-Nov-20 | 13-Jan-21 |
| | Region 4 | 482 448 | 16·3% | 238 | 55 | 23·1% | 27-Nov-20 | 18-Jan-21 |
| | Region 5 | 119 190 | 36·6% | 166 | 46 | 27·8% | 16-Nov-20 | 20-Jan-21 |
| | Region 6 | 768 446 | 20·7% | 240 | 154 | 64·3% | 26-Nov-20 | 21-Jan-21 |
| | Region 7 | 138 928 | 17·1% | 56 | 40 | 71·0% | 17-Nov-20 | 15-Dec-20 |
| | Tswane District | 3 709 635 | 23·3% | 1 606 | 946 | 58·9% | 06-Nov-20 | 21-Jan-21 |
| West Rand | Merafong City | 213 874 | 23·0% | 114 | 176 | 154·4% | 10-Nov-20 | 18-Dec-20 |
| | Mogale City | 435 254 | 19·7% | 164 | 255 | 155·3% | 09-Nov-20 | 17-Dec-20 |
| | Rand West City | 300 960 | 17·6% | 133 | 176 | 132·7% | 22-Nov-20 | 21-Dec-20 |
| | West Rand District | 950 088 | 20·0% | 411 | 607 | 147·8% | 09-Nov-20 | 21-Dec-20 |
| Unallocated | | | | | 748 | | | |
| GAUTENG PROVINCE | TOTAL | 15 176 113 | | 6025 | 6 332 | 108·4% | | |
| | | | 17·3% | | 5 584 | 92·7% | 04-Nov-20 | 22-Jan-21 |

Figure presents the target sample size and the achieved number of individuals. Across all sub-districts, the sample size was inflated by 25% to account for non-participation with 10% oversampling of households. The survey data collection was from November 4, 2020 to January 22, 2021.

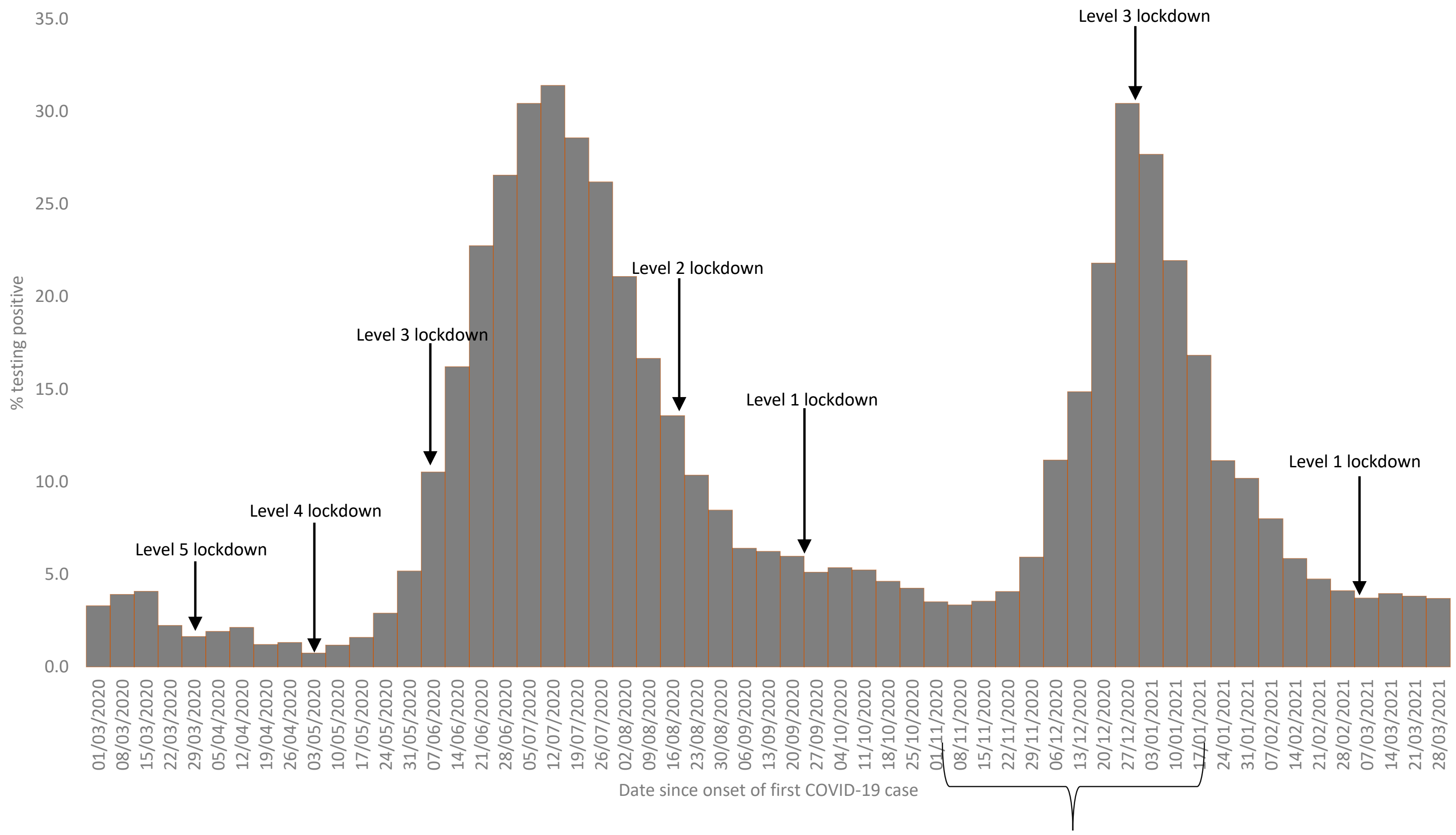


**Supplementary Figure S1: Sero-survey period by epidemic week showing weekly NAAT positivity rates.**

This figure depicts SARS-CoV-2 nucleic acid amplification test (NAAT) positivity trajectory since the first NAAT test in Gauteng, in relation to national outbreak control measures. NAAT testing positivity of >10% was used as a proxy for onset of a resurgence in Gauteng. The survey started November 4, 2020, 8 weeks after the end of the first wave (SARS-CoV-2 NAAT positivity had declined to <10% for the first wave) and coincided with the peak of the second wave. The last sampling was done on January 22, 2021, which was nine weeks after SARS-CoV-2 resurgence.

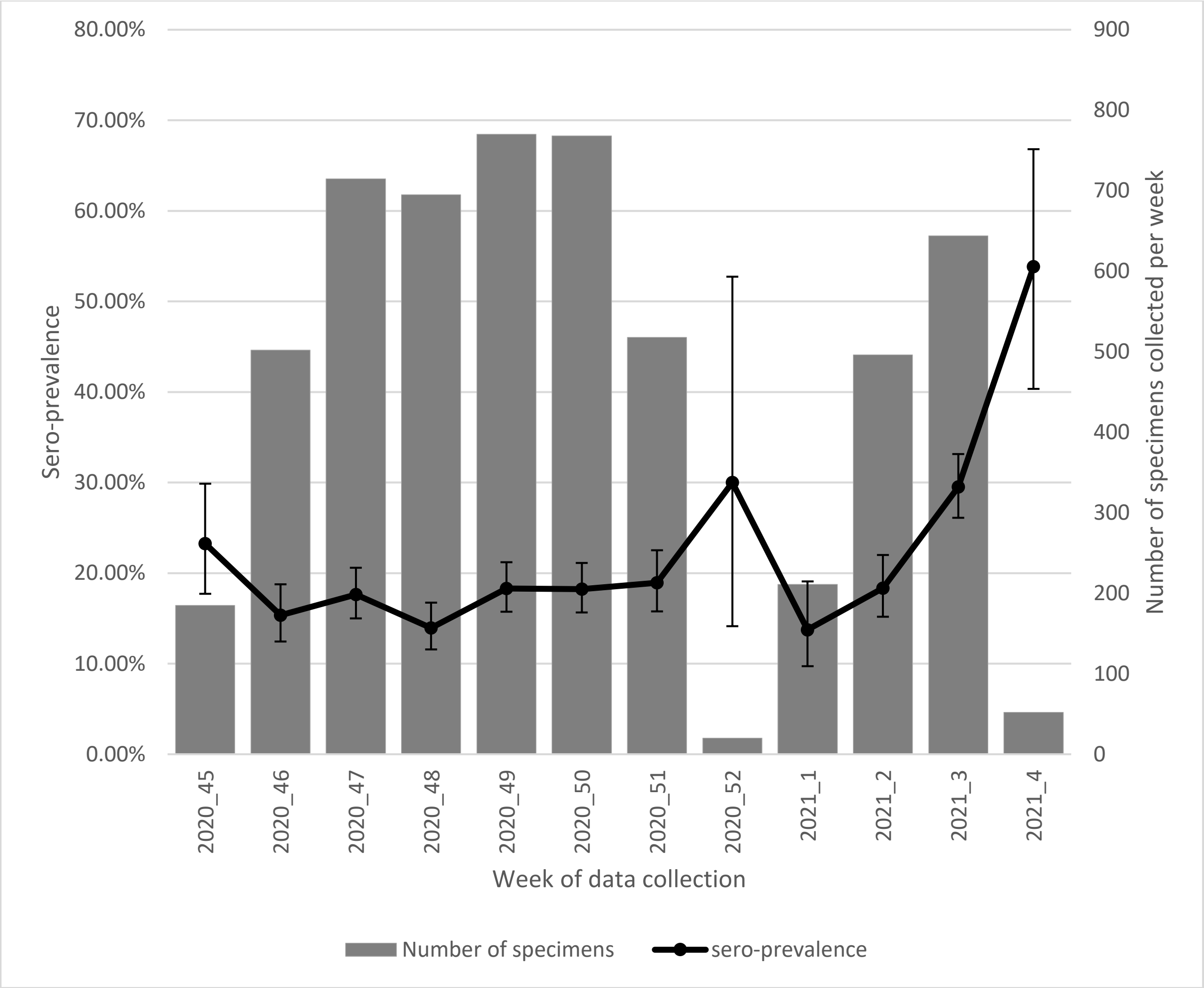


***Supplementary Figure S2: Number of specimens collected per week of survey data collection and the corresponding seroprevalence for specimens collected that week.***

We present the number of specimens collected per survey week and the corresponding seroprevalence for specimens collected that week. There was a significant increase in seroprevalence for specimens collected after the first week of January 2021.

## References

1. National Institute for Communicable Diseases. Surveillance Reports: weekly testing summary. https://www.nicd.ac.za/diseases-a-z-index/covid-19/surveillance-reports/weekly-testing-summary (accessed 30 March 2021); 2021.
2. Mendelson M, Madhi S. South Africa's COVID-19 testing strategy needs urgent fixing: Here's how to do it. https://theconversation.com/south-africas-covid-19-testing-strategy-needs-urgent-fixing-heres-how-to-do-it-138225 [Accessed 12 May 2020]: The Conversation; May 9 2020.
3. Jassat W, Cohen C, Mudara C, Blumberg L. Multivariable analysis comparing in-hospital mortality in the first and second wave of covid-19 in three districts of South Africa. *COVID-19 Special Public Health Surveillance Bulletin* 2021; **18**(6).
4. Stadlbauer D, Amanat F, Chromikova V, et al. SARS-CoV-2 Seroconversion in Humans: A Detailed Protocol for a Serological Assay, Antigen Production, and Test Setup. *Curr Protoc Microbiol* 2020; **57**(1): e100.
